# HELPING BEHAVIORS AND COGNITIVE FUNCTIONING IN LATER LIFE: A FOCUS ON DOSE AND ASYMMETRIC EFFECTS

**DOI:** 10.1093/geroni/igae098.0401

**Published:** 2024-12-31

**Authors:** Sae Hwang Han, Jeffrey Burr, Julia Tucker

**Affiliations:** University of Texas at Austin, Austin, Texas, United States; University of Massachusetts Boston, Boston, Massachusetts, United States; University of Texas at Austin, Austin, Texas, United States

## Abstract

Despite the burgeoning literature linking prosocial helping behaviors and cognitive functioning, empirical evidence indicating whether and how much ‘prescribing’ helping behaviors to older adults would confer cognitive benefits is limited. Further, earlier studies mostly focused on formal volunteering, while little is known about the cognitive benefits of informal helping given directly to non-household individuals. This study aims to bridge these gaps by identifying the cognitive benefits of transitioning into a formal volunteer and informal helper role, respectively, and to uncover the optimal dose-response parameters linking helping and cognition. Using longitudinal data from the Health and Retirement Study (1998–2020), we followed over 30,000 individuals aged 51 and older during the observation period spanning two decades. Cognitive functioning was assessed with the modified Telephone Interview for Cognitive Status (m-TICS; range: 0–27). Two forms of helping behaviors (i.e., formal volunteering and informal helping) were considered as status and time commitment. We used a multilevel modeling approach that allowed for estimating within–person effects specific to transitioning into (and out of) the respective helper role, as well as across different dosages of helping. Results indicated that transitioning into formal volunteering and informal helping were both associated with better cognitive functioning and slower cognitive decline. Further, moderate levels of helping (approximately 2–4 weekly hours) were consistently linked to robust cognitive benefits for both forms of helping. The study findings provide unique evidence supporting the view that social engagement through helping others in need represents modifiable lifestyle factors conducive to healthy cognitive aging.

